# *PARP-1 *Val762Ala polymorphism is associated with reduced risk of non-Hodgkin lymphoma in Korean males

**DOI:** 10.1186/1471-2350-11-38

**Published:** 2010-03-03

**Authors:** Xue Mei Jin, Hee Nam Kim, Il-Kwon Lee, Kyeong-Soo Park, Hyeoung-Joon Kim, Jin-Su Choi, Sang Woo Juhng, Chan Choi

**Affiliations:** 1 Genome Research Center for Hematopoietic Diseases, Chonnam National University Hwasun Hospital, 160, Ilsim-ni, Hwasun-eup, Hwasun-gun, Chonnam, 519-809, Republic of Korea; 2 Department of Pathology, Chonnam National University Medical School, 5 Hak-dong, Dong-gu, Gwangju, 501-746, Republic of Korea; 3 Department of Hematology/Oncology, Chonnam National University Medical School, 5 Hak-dong, Dong-gu, Gwangju, 501-746, Republic of Korea; 4 Department of Preventive Medicine, Chonnam National University Medical School, 5 Hak-dong, Dong-gu, Gwangju, 501-746, Republic of Korea; 5 Department of Preventive Medicine, College of Medicine, Seonam University, 720 Gwangchi-dong, Namwon, 590-711, Republic of Korea

## Abstract

**Background:**

Poly(ADP-ribose) polymerase-1 (PARP-1) is a nuclear enzyme that plays a role in DNA repair, differentiation, proliferation, and cell death. The polymorphisms of *PARP-1 *have been associated with the risk of various carcinomas, including breast, lung, and prostate. We investigated whether *PARP-1 *polymorphisms are associated with the risk of non-Hodgkin lymphoma (NHL).

**Methods:**

Subjects from a Korean population consisting of 573 NHL patients and 721 controls were genotyped for 5 *PARP-1 *polymorphisms (Asp81Asp, Ala284Ala, Lys352Lys, IVS13+118A>G, and Val762Ala) using High Resolution Melting polymerase chain reaction (PCR) and an automatic sequencer.

**Results:**

None of the 5 polymorphisms were associated with overall risk for NHL. However, the Val762Ala polymorphism was associated with reduced risk for NHL in males [odds ratio (OR), 0.62; 95% confidence interval (CI), 0.41-0.93 for CC genotype and OR, 0.84; 95% CI, 0.60-1.16 for TC genotype] with a trend toward a gene dose effect (p for trend, 0.02). The Asp81Asp (p for trend, 0.04) and Lys352Lys (p for trend, 0.03) polymorphisms revealed the same trend. In an association study of *PARP-1 *haplotypes, the haplotype-ACAAC was associated with decreased risk of NHL in males (OR, 0.75; 95% CI, 0.59-0.94).

**Conclusion:**

The present data suggest that Val762Ala, Asp81Asp, and Lys352Lys polymorphisms and the haplotype-ACAAC in *PARP-1 *are associated with reduced risk of NHL in Korean males.

## Background

Non-Hodgkin lymphoma (NHL) is the most common hematologic malignancy worldwide. It represents 4% of cancers, and is the fifth commonly diagnosed cancer in the United States [1]. Its rates are over 10/100,000 in the United States, Australia, and Western Europe, while less than 5/100,000 in Southern and Eastern Asia [2].

PARP-1 is a nuclear enzyme that catalyzes the poly(ADP-ribosyl)ation of target proteins in response to DNA damage; it is involved in DNA repair, cell death, proliferation, genome integrity, and modulation of gene transcription [3,4]. PARP-1 is involved in base excision reaction, which repairs DNA damage induced by chemical alterations, reactive oxidative species, and ionizing radiation. In addition, PARP-1 activation mediates apoptosis through the induction of translocation of apoptosis-inducing factor from the mitochondria to the nucleus [5].

The effect of PARP-1 on carcinogenesis is still unclear and controversial. PARP-1 activation has been reported to inhibit carcinogenesis by activation of DNA repair system [6-13]. *Parp *deficiencies have enhanced tumorigenesis and widened the tumor spectrum in *p53-*deficient mice [10]. Treatment with the alkylating agent, azoxymethane, enhanced the frequency of tumor development in the colon and liver of *Parp*-1^-/- ^mice [9]. Studies have demonstrated that *PARP-1 *Val762Ala polymorphism is associated with an increased risk of carcinomas, including prostate [8], esophagus [7], lung [12], stomach [11], breast [13], and urinary bladder [6]. In contrast, others have reported that inhibition or the absence of PARP-1 is associated with reduced risk of malignancy by inducing tumor cell apoptosis [14-18]. Loss of *Parp-1 *has increased tumor latency in *p53*-deficient mice [14]. *Parp-1*^-/- ^mice displayed a diminished susceptibility to skin carcinogenesis compared with *Parp-1*^+/+ ^mice after treatment with 7,12-dimethylbenz [a]-anthracene and 12-O-tetra-decanoyl-phorbol-13-acetate [17]. *PARP-1 *polymorphisms have been associated with reduced risk of malignancy in several case-control studies [15,16,18]. However, others have also proposed that PARP-1 has no effects on tumor development [19,20]. *Parp-1*^-/- ^mice did not demonstrate an increased incidence of tumor formation after either 4-nitroso-quinoline or 2-amino-3-methylimidazo treatment [19]. In a case-control study in Connecticut women, the risk of NHL was not associated with *PARP-1 *Val762Ala polymorphism [20].

Although many studies have searched for the association between PARP-1 polymorphisms and the risk of malignancy, the results are inconsistent in different organs and in different ethnic groups. We performed a population-based, case-control study to identify the association between *PARP-1 *polymorphisms and NHL risk in Korean subjects.

## Methods

### Study population

This case-control study included NHL patients (n = 573) and cancer-free controls (n = 721). All cases and controls were unrelated Korean individuals; the cases were adults (median age at diagnosis ± S.D 55.0 ± 14.6 years, range 15.0-90.0 years), histologically diagnosed with NHL between 1997 and 2006 at Chonnam National University Hospital (Gwangju, Korea) or Chonnam National University Hwasun Hospital (Hwasun, Korea) [21]. Lymph nodes or extranodal tissues were obtained and histologically diagnosed according to the World Health Organization (WHO) classification [22]. A staging workup included physical examination, laboratory examination, computed tomography scan, bone marrow biopsy, and lumbar puncture. Stages were defined according to the Ann Arbor system [23]. Types of NHL included diffuse large B-cell lymphomas (n = 330), T-cell lymphomas (n = 122), and other lymphomas (n = 121). The controls were frequency matched to patients by age within 5 years, sex, and county of residence. All of the healthy controls have no history of cancer (427 males, 304 females; median age ± S.D. 56.0 ± 15.3 years, range 17.0-82.5 years) (Table 1). All cases and controls provided informed consent for study participation. The study was approved by the Institutional Review Board of the Chonnam National University Hwasun Hospital in Hwasun, Korea.

**Table 1:** Frequency distribution of selected characteristics of study subjects According to case-control status

Characteristic	Cases	Controls	*P *value
No.(%)	573 (100)	721 (100)	
Age, no.(%)			
15-49 y	179 (31.2)	201 (27.9)	
50-64 y	226 (39.4)	286 (39.7	
≥ 65 y	168 (29.3)	234 (32.5)	0.33
Mean(yr.) ± SD	55.0 ± 14.6	56.0 ± 15.3	
Range	15.0-90.0	17.0-82.5	
Sex			
Male	336 (58.6)	417 (57.8)	
Female	237 (41.4)	304 (42.2)	0.77
NHL tumor pathology			
All B-cell lymphoma	451	NA	NA
Diffuse large B-cell	330	NA	NA
Marginal zone*	69	NA	NA
Follicular	17	NA	NA
Mantle cell	12	NA	NA
Small lymphocytic	8	NA	NA
Burkitt lymphoma	3	NA	NA
Others^B^	12	NA	NA
All T-cell lymphoma	122	NA	NA
Peripheral T-cell	55	NA	NA
Others^T^	67	NA	NA

*P *values for differences in socio-demographic characteristics between cases and controls.

NA indicates not applicable. Marginal zone* includes marginal zone B-cell lymphoma (n = 12) and mucosa-associated lymphoid tissue (MALT) (n = 57). Others^B ^includes B-cell prolymphocytic leukemia (n = 1) and undetermined B-cell lymphoma (n = 11). Others^T ^includes all T-cell lymphoma (n = 67) except for peripheral T-cell lymphoma.

### Genotyping

Genomic DNA was obtained from peripheral blood using a QIAamp DNA Blood Mini Kits (Qiagen, Valencia, CA, USA) according to the manufacturer's protocols. PCR cycling and high resolution melting (HRM) analysis was performed on the Rotor-Gene 6000™ (Corbett Research, Sydney, Australia) [24]. The PCR primers were as follows: for Val762Ala (86 bp fragment), 5'-taagtcgggggctttctttt-3' (forward), and 5'-agcagactgtaggccacctc-3' (reverse); for Asp81Asp (160 bp fragment), 5'-gatgggttctctgagcttcg-3' (forward), and 5'-gaggtttgctttgctctctga-3' (reverse); for Ala284Ala (76 bp fragment), 5'-gccctctgacatgtttctcc-3' (forward), and 5'-aaggagggcaccgaacac-3' (reverse); for Lys352Lys (178 bp fragment), 5'-caagggagagctggcttctt-3' (forward), and 5'-ggagttcacagcagcagga-3' (reverse); for IVS13+118A>G (124 bp fragment), 5'-tggatcaggtggcatcatag-3' (forward), and 5'-ggtactggccttcatgcaat-3' (reverse). The reaction mixture included genomic DNA (10 ng), 1× PCR buffer, 2.5 mM MgCl_2_, primers (100 nM), dNTPs (100 μM), SYTO 9 green fluorescent nucleic acid stain (2.5 μM; Invitrogen, Carlsbad, CA, USA), Taq. polymerase (0.5 U; Solgent, Daejeon, Korea) and water to a total volume of 10 μl. Cycling conditions included an initial 5 min hold at 95°C, followed by 40 cycles at 95°C for 5 s, annealing temperatures for 30 s, and 72°C for 20 s; a single cycle of 95°C for 1 s, 72°C for 90 s, and melting increasing from 70°C to 90°C at 0.1°C per second. Annealing temperatures for Asp81Asp, Ala284Ala, Lys352Lys, Val762Ala and IVS13+118A>G were 62°C, 56°C, 58°C, 56°C, and 60°C, respectively.

PCR products were column-purified after HRM analysis using the PCR-M clean up kit (GeneAll Biotechnology, Seoul, Korea) according to the manufacturer's instructions. PCR products were eluted in a 20 μl volume, purified PCR products were used as templates in the Big Dye Terminator v3.1 Cycling Sequencing kit (Applied Biosystems, Foster City, CA, USA). The reaction mix consisted of 1× terminator pre-mix, 1× sequencing buffer, primers (667 nM), and purified template (1 μl) in a 10 μl total volume. Reactions were run on a PTC-100 thermal cycler (MJ Research, Reno, NV, USA) as follows: a single cycle of 95°C for 1 min, 25 cycles of 95°C for 10 s, 50°C for 5 s, and 60°C for 4 min. Sequencing reactions were ethanol-precipitated and run on a 3100 Genetic Analyser (Applied Biosystems). In 90 subjects, the results of HRM analyses were compared with those from cycle sequencing, and the resulting concordance rate was 100%.

### Statistical analysis

Hardy-Weinberg equilibrium testing was performed using the chi-square goodness-of-fit test. The Pearson chi-square test was used to examine the genotype distribution differences between cases and controls. Adjusted odds ratios (ORs) were calculated using a logistic regression model that controlled for gender and age and included 95% confidence intervals (CIs). Statistical significance was defined at *p *values less than 0.05, and all statistical tests were 2-sided. Haplotypes and haplotype frequencies were calculated using the SNPAnalyzer-Pro version 1.8 software (Istech, Goyang, Korea) to analyze associations between haplotypes and NHL risk after stratification by age at diagnosis, sex, and NHL subtype. All calculations were performed using an SPSS software package, version 13.0 (SPSS, Chicago, IL). *PARP-1 *inter-SNP linkage disequilibrium was calculated using Haploview software version 4.1 http://www.broad.mit.edu/mpg/haploview for the set of control samples (n = 721).

## Results

**Figure 1:**
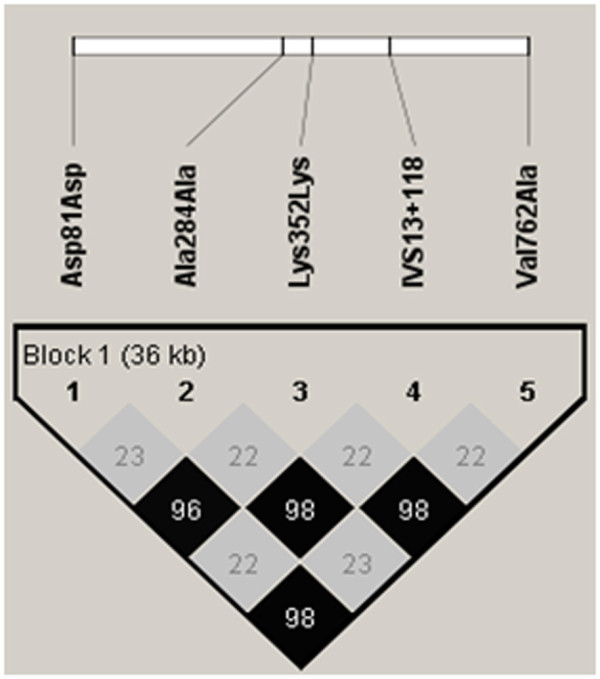
***PARP-1 *inter-polymorphism linkage disequilibrium**. *PARP-1 *inter-polymorphism linkage disequilibrium. r^2 ^between pairs of polymorphisms was calculated based on all the controls using Haploview v4.1 http://www.broad.mit.edu/mpg/haploview.

Characteristics of the study population are summarized in Table 1. There were no differences in the frequency distribution for age (*p *= 0.33) or sex (*p *= 0.77) between cases and controls. The control genotype distributions were in agreement with the Hardy-Weinberg equilibrium (*p *= 0.61 for Asp81Asp, *p *= 0.31 for Ala284Ala, *p *= 0.83 for Lys352Lys, *p *= 0.38 for IVS13+118 A>G, *p *= 0.84 for Val762Ala). Haploview version 4.1 determined that the *PARP-1 *locus contained 1 block of linkage disequilibrium (Figure. 1). Two groups of polymorphisms were present in the block; one group consisted of Asp81Asp, Lys352Lys, and Val762Ala polymorphism, and the other included Ala284Ala and IVS13+118A>G polymorphism. Both revealed high intra-group linkage disequilibrium (r^2^, ≥ 0.96), but low inter-group linkage disequilibrium (r^2^, 0.22-0.23).

Table 2 summarizes the association study between the 5 polymorphisms and the overall risk for NHL. Analysis revealed no significant associations between polymorphisms and overall NHL risk. Neither the CC genotype of Val762Ala (OR, 0.85; 95%CI, 0.62-1.17) nor the TC genotype (OR, 0.93; 95%CI, 0.72-1.19) were associated with risk for NHL. The genotypes of the remaining 4 polymorphisms were also associated with risk for NHL.

**Table 2:** Association testing of *PARP-1 *polymorphisms with risk of NHL

NPs	Genotype	Controls no.(%)	Cases no.(%)	OR (95% CI)
Asp81Asp	GG	227 (31.5)	185 (32.5)	1
	GA	349 (48.4)	279 (49.0)	0.99 (0.77-1.27)
	AA	145 (20.1)	105 (18.5)	0.90 (0.65-1.24)
	GA+AA	494 (68.5)	384 (67.5)	0.96 (0.76-1.21)
				
Ala284Ala	CC	424 (58.8)	357 (62.4)	1
	CT	264 (36.6)	189 (33.0)	0.85 (0.67-1.07)
	TT	33 (4.6)	26 (4.5)	0.94 (0.55-1.60)
	CT+TT	297 (41.2)	215 (37.6)	0.86 (0.69-1.08)
				
Lys352Lys	GG	222 (30.8)	184 (32.1)	1
	GA	353 (49.0)	285 (49.7)	0.98 (0.76-1.26)
	AA	145 (20.1)	104 (18.2)	0.87 (0.64-1.20)
	GA+AA	498 (69.2)	389 (67.9)	0.95 (0.75-1.20)
				
IVS13+118 A>G	AA	426 (59.2)	361 (63.0)	1
	AG	261 (36.3)	188 (32.8)	0.85 (0.67-1.07)
	GG	33 (4.6)	24 (4.2)	0.86 (0.50-1.48)
	AG+GG	294 (40.8)	212 (37.0)	0.85 (0.68-1.06)
				
Val762Ala	TT	221 (30.7)	189 (33.0)	1
	TC	354 (49.1)	279 (48.7)	0.93 (0.72-1.19)
	CC	146 (20.2)	105 (18.3)	0.85 (0.62-1.17)
	TC+CC	500 (69.3)	384 (67.0)	0.90 (0.71-1.14)

All analyses have been age- and sex-adjusted. *P *values were > 0.05 in all analyses.

As presented in Additional file 1: table S1, males with the TC genotype of Val762Ala demonstrated a slightly reduced NHL risk (OR, 0.84; 95% CI, 0.60-1.16) compared to those with the TT genotype. And the males with the CC genotype had a significantly reduced risk (OR, 0.62; 95% CI, 0.41-0.93) which revealed a trend toward a gene dose effect (p for trend, 0.02). However, there was no association between Val762Ala polymorphism and NHL risk in females. There was significant interaction seen between sex and the Val762Ala polymorphism (p for interaction, 0.048). Asp81Asp (p for trend, 0.04) and Lys352Lys (p for trend, 0.03) polymorphisms revealed similar trends, although interactions between sex and polymorphism were a borderline significance levels (p for interaction of Asp81Asp, 0.062; p for interaction of Lys352Lys, 0.067). However, *PARP-1 *polymorphisms did not show associations with the NHL subtypes (Additional file 1: table S2) or age (data not shown).

The distribution of *PARP-1 *haplotype frequencies and association of haplotypes with risk for NHL are summarized in Additional file 1: table S3. Three common haplotypes (ACAAC, GCGAT, and GTGGT) and several rare ones (with frequency < 5%) were inferred, and rare haplotypes were excluded in the association analyses. None of the haplotypes were associatied with overall risk for NHL. However the haplotype-ACAAC in males was associated with decreased risk for NHL (OR, 0.74; 95% CI, 0.59-0.94) when haplotype-GCGAT was used as a reference.

## Discussion

We investigated the association between 5 *PARP-1 *polymorphisms and NHL risk in a Korean population. Three polymorphisms (Asp81Asp, Lys352Lys, and Val762Ala) were associated with decreased risk of NHL in males. A association testing of haplotype-ACAAC revealed a similar result. However, none of the *PARP-1 *polymorphisms or haplotypes were associated with overall NHL risk.

Our data appear to support the hypothesis that accumulated DNA damage may lead to enhanced apoptosis during cell division, which could result in protection from development of malignancy with intact apoptotic mechanisms. Concordant findings with the results of this study have been identified in case-control studies of squamous cell carcinoma of the head and neck [15], breast carcinoma [18], and adult glioma [16]. In animal experiments, overexpression of dominant negative PARP-1 prevented *in vivo *tumor formation by HeLa cells in nude mice due to increased apoptosis of tumor cells [25].

*PARP-1 *polymorphisms were associated with decreased risk of NHL in males in this study. The sexual dimorphism might be associated with the female sex hormone, 17-β-estradiol. PARP strongly interacts with estrogen receptor α and DNA in the presence of 17-β-estradiol, which leads to inhibition of PARP activation [26]. Sex-dependent response to PARP activation has also been reported in an endotoxin-induced inflammation and vascular change model, female mice were more resistant to endotoxin than male mice[26]. In a neonatal-stroke mouse model, disruption of the *PARP-1 *gene selectively protected male mice against brain injury [27]. Also in an adult-stroke mouse model, female neuronal nitric oxide synthase knockout (nNOS-/-) mice were more severely damaged after middle cerebral artery occlusion than wild-type females. In addition, male nNOS-/- littermates were protected [28]. In a case-control study, *PARP-1 *polymorphism has been associated with reduced risk of adult glioma in men [16].

Inconsistency of the association between *PARP-1 *polymorphism and malignancy risk might result from differences in genetic background, environmental factors, organ specificity, and study sample size. For example, the frequency of the Val762Ala genetic polymorphism differs between ethnic groups. In the present study of Koreans, the frequency of the C allele is 0.448 which is consistent with that observed in previous studies of Koreans (0.444) and Han Chinese (0.389) [12,29]. However, the frequency is 0.145 in Caucasian-Americans and 0.045 among African-Americans [8]. PARP-1 expression levels vary in carcinomas of different organs. It is high in lymphoma [30] and endometrial carcinoma [31], and low in breast carcinoma [32,33] and laryngeal carcinoma [34].

In this present study, haplotype-ACAAC was also associated with reduced risk of NHL in males, although there was no association between any haplotypes and overall NHL risk. It agrees with the results that the A allele of Asp81Asp, A allele of Lys352Lys, and C allele of Val762Ala are significantly associated with decreased risk of NHL.

As the sample size of this study is not sufficiently large and is restricted to Korean population, the present data should be validated in larger samples and in other ethnic groups. Although *PARP-1 *polymorphisms are associated with decreased risk of NHL in the present study, we could not exclude the possibility that other un-typed variants located in or near the *PARP-1 *locus might be associated with reduced risk of NHL. Additional surveys examining other variants around the locus are required to resolve this issue. A gene-gene interaction study examining genes involved in apoptosis or DNA repair might allow for a more comprehensive perspective.

## Conclusion

In a polulation-based, case-control study to identify the association between polymorphisms in *PARP-1 *and NHL risk in Koreans, we found that Val762Ala, Asp81Asp, and Lys352Lys polymorphisms and the haplotype-ACAAC in *PARP-1 *were associated with decreased risk of NHL in males.

## Competing interests

The authors declare that they have no competing interests.

## Authors' contributions

All authors read and approved the final version to be published. XMJ carried out genotyping and drafted the manuscript. HNK participated in sequence alignment. IKL involved in drafting the manuscript and revising it critically. KSP did statistical analysis of the data. HJK have given final approval of the version to be published. JSC collected the control samples and advised in data analysis. SWJ designed of the study. CC designed the study and helped to draft the manuscript.

## Pre-publication history

The pre-publication history for this paper can be accessed here:

http://www.biomedcentral.com/1471-2350/11/38/prepub

## Supplementary Material

Additional file 1 Supplemental tables S1, S2 and S3. Click here for file
