# Supplementary material for: PARP-1 Val762Ala polymorphism is associated with reduced risk of non-Hodgkin lymphoma in Korean males
Source: BMC Med Genet. 2010 Mar 3;11:38. doi: 10.1186/1471-2350-11-38 (PMC2843603; doi:10.1186/1471-2350-11-38)
Supplement: Additional file 1 — Supplemental tables S1, S2 and S3. [file 1471-2350-11-38-S1.DOCX]

Additional file 1

Table S1.. Association tests of *PARP-1* polymorphisms with the risk of NHL by sex

|  |  | Genotype | | | | |  |  |  |  |  |  |  |
| --- | --- | --- | --- | --- | --- | --- | --- | --- | --- | --- | --- | --- | --- |
|  | Control No.(%) | | |  | NHL No.(%) | | |  | OR (95% CIs) | | | *P*^†^ | P^‡^ |
|  |  |  |  |  |  |  |  |  |  |  |  |  |  |
| Asp81Asp | GG | GA | AA |  | GG | GA | AA |  | GG | GA | AA |  |  |
| Male | 126 (30.2) | 197 (47.2) | 94 (22.5) |  | 116 (34.8) | 161 (48.3) | 56 (16.8) |  | 1 | 0.90 (0.65-1.26) | 0.66 (0.44-1.00) | 0.04 |  |
| Female | 101 (33.2) | 152 (50.0) | 51 (16.8) |  | 69 (29.2) | 118 (50.0) | 49 (20.8) |  | 1 | 1.13 (0.77-1.67) | 1.40 (0.85-2.30) | 0.19 | 0.062 |
|  |  |  |  |  |  |  |  |  |  |  |  |  |  |
| Ala284Ala |  |  |  |  |  |  |  |  |  |  |  |  |  |
| Male | 256 (61.4) | 141 (33.8) | 20 (4.8) |  | 208 (62.1) | 111 (33.1) | 16 (4.8) |  | 1 | 0.97 (0.71-1.32) | 0.98 (0.50-1.95) | 0.87 |  |
| Female | 168 (55.3) | 123 (40.5) | 13 (4.3) |  | 149 (62.9) | 78 (32.9) | 10 (4.2) |  | 1 | 0.72 (0.50-1.03) | 0.86 (0.37-2.02) | 0.13 | 0.45 |
|  |  |  |  |  |  |  |  |  |  |  |  |  |  |
| Lys352Lys | GG | GA | AA |  | GG | GA | AA |  | GG | GA | AA |  |  |
| Male | 123 (29.6) | 199 (47.8) | 94 (22.6) |  | 113 (33.6) | 168 (50.0) | 55 (16.4) |  | 1 | 0.93 (0.67-1.30) | 0.65 (0.43-0.99) | 0.03 |  |
| Female | 99 (32.6) | 154 (50.7) | 51 (16.8) |  | 71 (30.0) | 117 (49.4) | 49 (20.7) |  | 1 | 1.06 (0.72-1.56) | 1.33 (0.81-2.19) | 0.28 | 0.067 |
|  |  |  |  |  |  |  |  |  |  |  |  |  |  |
| IVS13+118 A>G |  |  |  |  |  |  |  |  |  |  |  |  |  |
| Male | 258 (61.9) | 139 (33.3) | 20 (4.8) |  | 212 (63.1) | 110 (32.7) | 14 (4.2) |  | 1 | 0.96 (0.71-1.31) | 0.84 (0.41-1.71) | 0.66 |  |
| Female | 168 (55.4) | 122 (40.3) | 13 (4.3) |  | 149 (62.9) | 78 (32.9) | 10 (4.2) |  | 1 | 0.73 (0.51-1.04) | 0.86 (0.37-2.02) | 0.14 | 0.48 |
|  |  |  |  |  |  |  |  |  |  |  |  |  |  |
| Val762Ala | TT | TC | CC |  | TT | TC | CC |  | TT | TC | CC |  |  |
| Male | 121 (29.0) | 201 (48.2) | 95 (22.8) |  | 118 (35.1) | 162 (48.2) | 56 (16.7) |  | 1 | 0.84 (0.60-1.16) | 0.62 (0.41-0.93) | 0.02 |  |
| Female | 100 (32.9) | 153 (50.3) | 51 (16.8) |  | 71 (30.0) | 117 (49.4) | 49 (20.7) |  | 1 | 1.07 (0.73-1.58) | 1.34 (0.81-2.20) | 0.26 | 0.048 |

All analyses have been adjusted by age.

P^†^, value for trend.

P^‡^ , value for interaction between sex and *PARP-1* genotypes.

Table S2. Association tests of *PARP-1* polymorphisms with the risk of NHL subtypes

|  |  | All B-cell lymphoma | |  | DLBCL | |  | All T-cell lymphoma | |
| --- | --- | --- | --- | --- | --- | --- | --- | --- | --- |
|  | Genotype | Patients, no.(%) | OR (95% CI) |  | Patients,no.(%) | OR (95% CI) |  | Patients, no.(%) | OR (95% CI) |
| Asp81Asp | GG | 147 (32.9) | 1 |  | 101 (31.0) | 1 |  | 38 (31.1) | 1 |
|  | GA | 213 (47.7) | 0.94 (0.72-1.23) |  | 151 (46.3) | 0.97 (0.72-1.32) |  | 66 (54.1) | 1.16 (0.75-1.79) |
|  | AA | 87 (19.5) | 0.93 (0.66-1.30) |  | 74 (22.7) | 1.15 (0.80-1.65) |  | 18 (14.8) | 0.77 (0.42-1.40) |
|  |  |  |  |  |  |  |  |  |  |
| Ala284Ala | CC | 282 (62.7) | 1 |  | 208 (63.2) | 1 |  | 75 (61.5) | 1 |
|  | CT | 143 (31.8) | 0.81 (0.63-1.05) |  | 102 (31.0) | 0.79 (0.59-1.05) |  | 46 (37.7) | 0.98 (0.66-1.46) |
|  | TT | 25 ( 5.6) | 1.14 (0.66-1.96) |  | 19 ( 5.8) | 1.17 (0.65-2.11) |  | 1 ( 0.8) | 0.17 (0.02-1.27) |
|  |  |  |  |  |  |  |  |  |  |
| Lys352Lys | GG | 147 (32.6) | 1 |  | 102 (30.9) | 1 |  | 37 (30.3) | 1 |
|  | GA | 217 (48.1) | 0.93 (0.71-1.21) |  | 154 (46.7) | 0.95 (0.70-1.28) |  | 68 (55.7) | 1.17 (0.76-1.82) |
|  | AA | 87 (19.3) | 0.91 (0.65-1.27) |  | 74 (22.4) | 1.11 (0.77-1.60) |  | 17 (13.9) | 0.73 (0.39-1.34) |
|  |  |  |  |  |  |  |  |  |  |
| IVS13+118 | AA | 286 (63.4) | 1 |  | 210 (63.6) | 1 |  | 75 (61.5) | 1 |
| A>G | AG | 142 (31.5) | 0.81 (0.63-1.04) |  | 102 (30.9) | 0.79 (0.60-1.05) |  | 46 (37.7) | 0.99 (0.66-1.48) |
|  | GG | 23 ( 5.1) | 1.04 (0.60-1.81) |  | 18 ( 5.5) | 1.11 (0.61-2.01) |  | 1 ( 0.8) | 0.17 (0.02-1.27) |
|  |  |  |  |  |  |  |  |  |  |
| Val762Ala | TT | 150 (33.3) | 1 |  | 105 (31.8) | 1 |  | 39 (32.0) | 1 |
|  | TC | 214 (47.5) | 0.89 (0.68-1.16) |  | 152 (46.1) | 0.90 (0.67-1.22) |  | 65 (53.3) | 1.06 (0.69-1.63) |
|  | CC | 87 (19.3) | 0.88 (0.63-1.23) |  | 73 (22.1) | 1.05 (0.73-1.52) |  | 18 (14.8) | 0.72 (0.40-1.32) |

DLBC, diffuse large B cell-Lymphoma,

OR adjusted by age and sex.

*P* value was > 0.05 in all analyses.

Table S3. Association tests of haplotypes of *PARP-1* polymorphisms with the risk of NHL by sex

|  |  |  |  | Haplotypes No.(%) | | |  |  |  |  |  |  |  |
| --- | --- | --- | --- | --- | --- | --- | --- | --- | --- | --- | --- | --- | --- |
|  |  |  | Controls |  |  |  | Cases |  |  | OR (95%CI) | | |  |
|  |  | GCGAT | GTGGT | ACAAC |  | GCGAT | GTGGT | ACAAC |  | GCGAT | GTGGT | ACAAC |  |
| All subjects | | 472 (33.1) | 319 (22.4) | 634 (44.5) |  | 406 (36.3) | 232 (20.8) | 479 (42.9) |  | 1 | 0.85 (0.68-1.05) | 0.88 (0.74-1.05) |  |
| Sex | |  |  |  |  |  |  |  |  |  |  |  |  |
|  | Male | 267 (32.4) | 174 (21.1) | 382 (46.4) |  | 247 (38.4) | 133 (20.7) | 263 (40.9) |  | 1 | 0.83 (0.62-1.10) | 0.74 (0.59-0.94) |  |
|  | Female | 205 (34.2) | 144 (24.0) | 251 (41.8) |  | 157 (33.5) | 98 (20.9) | 213 (45.5) |  | 1 | 0.89 (0.64-1.24) | 1.11 (0.84-1.46) |  |

Each subject contributes two haplotypes to the analysis, thus the total number of haplotypes in each colum is twice the number of subjects.

The order of the polymorphism is as follows: Asp81Asp, Ala284Ala, Lys352Lys, IVS13+118A>G, and Val762Ala.

P value for haplotype-ACAAC of male was 0.01. However, all of p values for other haplotypes of male and female was >0.05.

The haplotypes with allele frequency <5% were excluded.
